# Glucocorticoids target the CXCL9/CXCL10-CXCR3 axis and confer protection against immune-mediated kidney injury

**DOI:** 10.1172/jci.insight.160251

**Published:** 2023-01-10

**Authors:** Jan-Hendrik Riedel, Lennart Robben, Hans-Joachim Paust, Yu Zhao, Nariaki Asada, Ning Song, Anett Peters, Anna Kaffke, Alina Borchers, Gisa Tiegs, Larissa Seifert, Nicola M. Tomas, Elion Hoxha, Ulrich O. Wenzel, Tobias B. Huber, Thorsten Wiech, Jan-Eric Turner, Christian F. Krebs, Ulf Panzer

**Affiliations:** 1 Division of Translational Immunology, III. Department of Medicine and; 2 III. Department of Medicine, University Medical Center Hamburg-Eppendorf, Hamburg, Germany.; 3 Institute of Medical Systems Biology, Center for Molecular Neurobiology Hamburg (ZMNH), Hamburg, Germany.; 4 Institute of Experimental Immunology and Hepatology,; 5 Institute of Pathology, Section of Nephropathology, and; 6 Hamburg Center for Translational Immunology, University Medical Center Hamburg-Eppendorf, Hamburg, Germany.

**Keywords:** Immunology, Nephrology, Autoimmune diseases, Chemokines, T cells

## Abstract

Glucocorticoids remain a cornerstone of therapeutic regimes for autoimmune and chronic inflammatory diseases — for example, in different forms of crescentic glomerulonephritis — because of their rapid antiinflammatory effects, low cost, and wide availability. Despite their routine use for decades, the underlying cellular mechanisms by which steroids exert their therapeutic effects need to be fully elucidated. Here, we demonstrate that high-dose steroid treatment rapidly reduced the number of proinflammatory CXCR3^+^CD4^+^ T cells in the kidney by combining high-dimensional single-cell and morphological analyses of kidney biopsies from patients with antineutrophil cytoplasmic antibody–associated (ANCA-associated) crescentic glomerulonephritis. Using an experimental model of crescentic glomerulonephritis, we show that the steroid-induced decrease in renal CD4^+^ T cells is a consequence of reduced T cell recruitment, which is associated with an ameliorated disease course. Mechanistic in vivo and in vitro studies revealed that steroids act directly on renal tissue cells, such as tubular epithelial cells, but not on T cells, which resulted in an abolished renal expression of CXCL9 and CXCL10 as well as in the prevention of CXCR3^+^CD4^+^ T cell recruitment to the inflamed kidneys. Thus, we identified the CXCL9/CXCL10-CXCR3 axis as a previously unrecognized cellular and molecular target of glucocorticoids providing protection from immune-mediated pathology.

## Graphical abstract

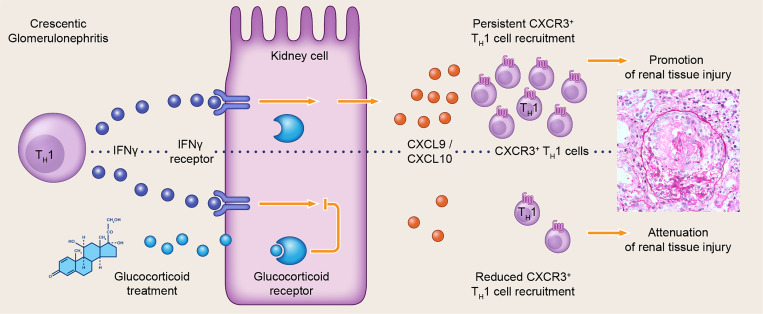

## Introduction

Immune-mediated kidney diseases remain a leading cause of end-stage kidney failure worldwide. The recruitment of immune cells into the kidney is a morphological hallmark of this group of disorders that is closely correlated to the clinical outcome (1, 2). In particular, in rapidly progressive or crescentic glomerulonephritis (RPGN), infiltrating CD4^+^ effector T cells of the Th1 and Th17 types release proinflammatory cytokines that directly promote tissue damage and stimulate chemokine production by renal resident cells, leading to the recruitment of additional leukocyte subsets and the subsequent loss of renal function, while the role of CD8^+^ T cells in RPGN remains controversial (3–7).

In experiments giving rise to this study, we observed that steroid treatment markedly reduced the renal T cell infiltrate in patients with antineutrophil cytoplasmic antibody–associated (ANCA-associated) crescentic GN, the leading cause of RPGN. Further analyses revealed that, particularly, the number of effector CD4^+^ T cells, not CD8^+^ T cells, was diminished. This finding prompted our interest in investigating the underlying mechanisms of how steroids induce these therapeutic effects on CD4^+^ T cells.

Glucocorticoids (GCs), a class of lipid-soluble, cholesterol-derived corticosteroid hormones (8), still serve as the cornerstone of frontline therapeutic regimes for autoimmune and chronic inflammatory disorders, including immune-mediated glomerular diseases, owing to their potent and rapid immunosuppressive and antiinflammatory properties (9). The reliance on nonspecific immunosuppression is often associated with severe infectious complications. Besides, the use of GCs entails a wide variety of significant adverse effects in most patients (10). GCs primarily function through binding to the cytosolic GC receptor (GR), expressed in virtually every nucleated cell; the cytosolic GR then translocates into the nucleus interacting with GC-responsive elements of the DNA to activate or suppress transcription (11). However, despite their long-standing use in a wide range of inflammatory diseases, the exact mechanisms of action of GCs need to be addressed in full — both those producing beneficial and those causing adverse effects. It is especially essential to develop new therapies with fewer adverse events.

Accumulating evidence suggests a cell type– and tissue-specific responsiveness to GC therapy that is even more complex due to acute and chronic effects, the different types of GCs, and the dose used in clinical applications (10). Certainly, T cells are thought to be primary targets of GC therapy. However, conflicting evidence exists concerning their responsiveness to GCs, such as cell death and proliferation, which may partially be explained by the subtypes of T cells reacting differently to GC exposure (8). Moreover, the effects of GCs on other leukocyte subjects, such as macrophages and neutrophils (12), and resident cells, such as epithelial cells (13), are not characterized as well and may have been underrated in previous studies.

The aim of the present study was to investigate the effects of a high-dose GC pulse regimen reflecting the standard of care for remission induction of ANCA-GN on the renal leukocyte infiltrate in human and experimental crescentic glomerulonephritis. Here, we (a) assessed the direct effects of GCs on the renal T cell infiltrate in human and murine crescentic glomerulonephritis (cGN) by combining high-dimensional, single-cell and morphological analyses; (b) dissected the observed effects in terms of T cell intrinsic versus tissue-specific causes; and (c) identified cellular and molecule targets responsible for the GC-mediated regulation of the renal T cell infiltrate in human and experimental cGN.

## Results

### GC treatment rapidly reduced the renal CD4^+^ T cell infiltrate in human ANCA-GN.

**Figure 1:**
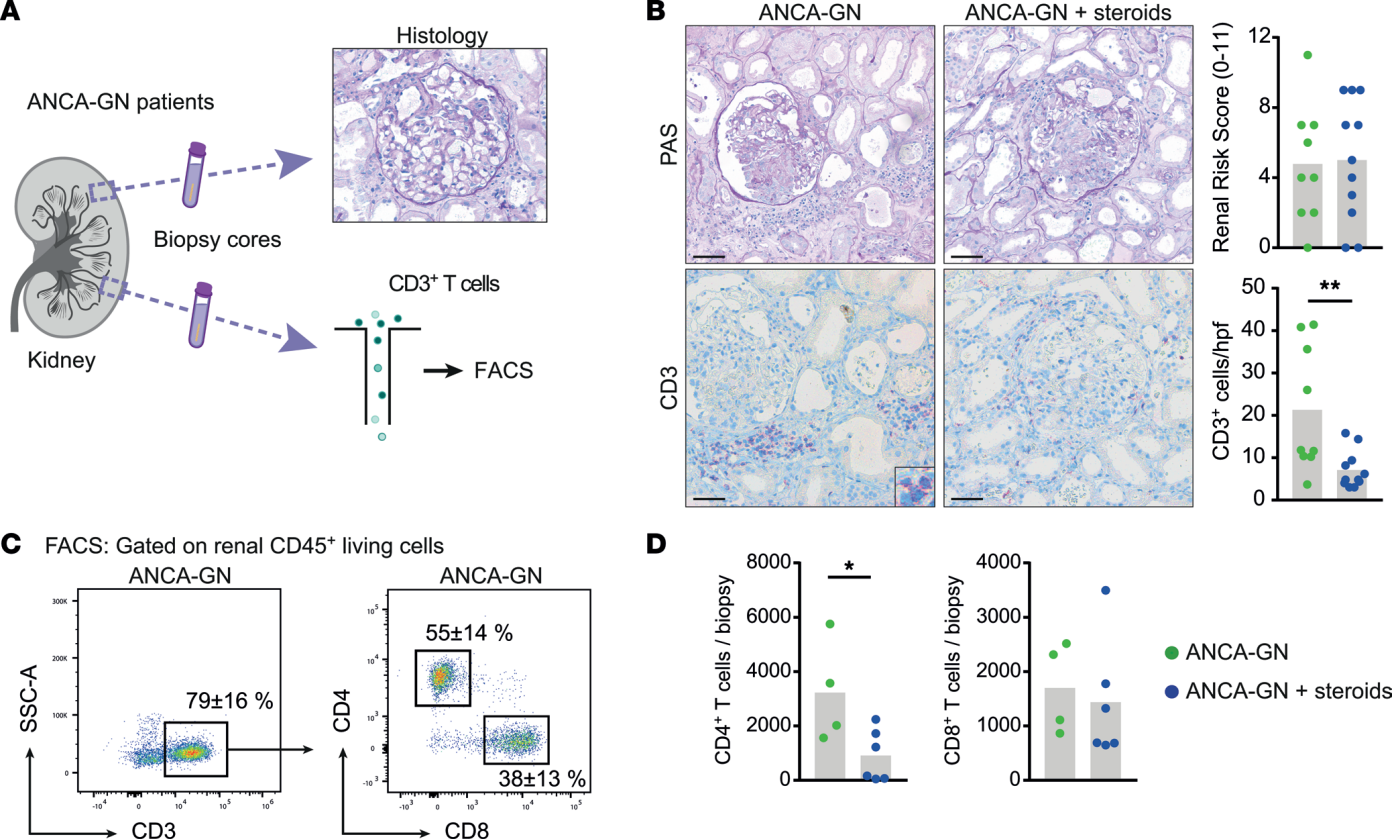
Glucocorticoid treatment rapidly reduces the renal CD4^+^ T cell infiltrate in human ANCA-GN. (**A**) Schematic representation of the experimental setup. (**B**) Representative photographs of PAS-stained kidney sections and the respective Renal Risk Score (14) of kidney biopsies obtained from untreated patients with ANCA-GN and patients with ANCA-GN after glucocorticoid treatment, as well as representative photographs of serial immunohistochemical CD3 staining and semiquantitative analysis of intrarenal T cell numbers. (**C**) Representative flow cytometric plots showing mean percentages of CD3^+^ cells of total CD45^+^ cells and mean percentages of CD3^+^CD4^+^ and CD3^+^CD8^+^ T cells of total CD3^+^ T cells in patients with ANCA-GN. (**D**) Quantification of total renal CD3^+^CD4^+^ and CD3^+^CD8^+^ T cells of patients with ANCA-GN with and without steroid treatment. Scale bar: 25 μm. Symbols represent individual data points, with the mean as a bar graph. Data were analyzed using a 2-tailed *t* test. *P < 0.05, **P < 0.01.

For a systematic analysis of T cells from patients with cGN, we established a workflow designed to generate histological and single-cell data from a pool of patients with biopsy-proven ANCA-GN (Figure 1A). We were able to recruit almost equal numbers of patients with ANCA-GN who had been treated with 1 up to 3 pulses of i.v. GCs (ANCA-GN + steroids) and patients who had not been treated (ANCA-GN) before kidney biopsy but had comparable baseline characteristics (Supplemental Figure 1; supplemental material available online with this article; https://doi.org/10.1172/jci.insight.160251DS1). periodic acid–Schiff (PAS) staining revealed similar levels of tissue injury in both groups (Figure 1B). Furthermore, a clinicopathological score designed to predict renal outcomes in patients with ANCA-GN (14) showed a similar distribution, ruling out a selection bias toward healthier patients in the treatment-naive group. The high-dose i.v. GC treatment significantly reduced the number of CD3^+^ T cells determined by semiquantitative analysis of IHC CD3^+^ staining (Figure 1B). This was corroborated by flow cytometric data of intrarenal T cells gathered from kidney biopsy material of patients with ANCA-GN. Analyses revealed CD3^+^CD4^+^ T cells as the main T cell subtype in the inflamed kidneys (Figure 1C), with absolute CD3^+^CD4^+^ T cell numbers significantly reduced in patients with ANCA-GN treated with GCs, while CD3^+^CD8^+^ T cells were only marginally decreased (Figure 1, C and D).

### GCs decrease the CD4^+^ T cell number in the kidney in an experimental model of cGN.

**Figure 2:**
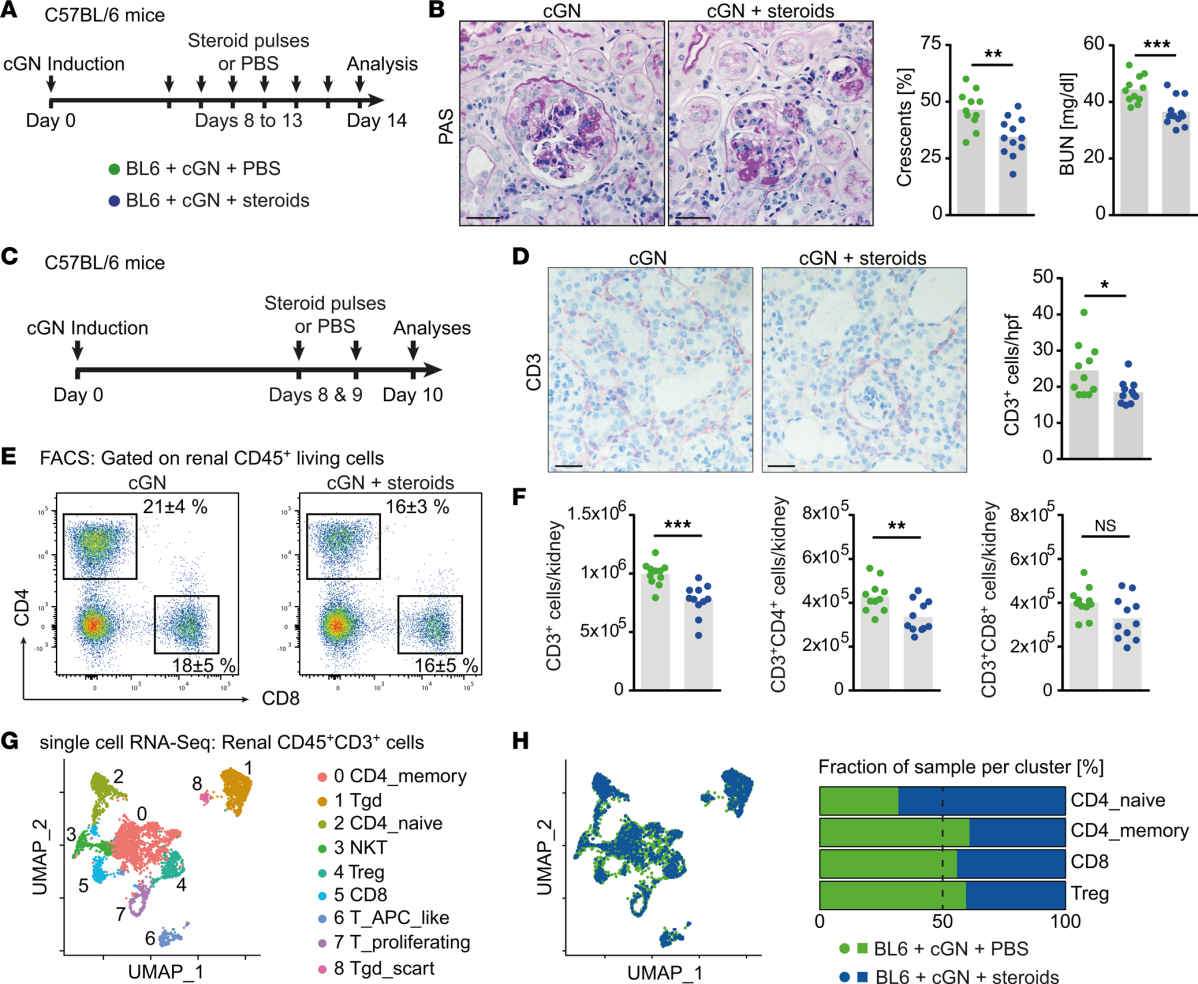
Glucocorticoids reduce the renal CD4^+^ T cell infiltrate in murine cGN. (**A**) Schematic representation of the experimental setup. (**B**) Representative photographs of PAS-stained kidney sections and quantification of crescent formation and BUN levels of untreated nephritic mice and mice treated for 6 consecutive days with high doses of steroids. (**C**) Schematic representation of the experimental setup. (**D**) Representative photographs of immunohistochemical CD3 staining of untreated and steroid-treated nephritic mice and semiquantitative analysis of intrarenal T cell numbers. (**E**) Representative plots showing mean percentages of renal CD4^+^ and CD8^+^ T cells. (**F**) Quantification of flow cytometric analyses of changes in CD3^+^, CD3^+^CD4^+^, and CD3^+^CD8^+^ T cells performed for the previously mentioned groups. (**G**) Uniform Manifold Approximation and Projection (UMAP) space of 9 clusters defined by unsupervised clustering of single-cell RNA-Seq of renal CD3^+^ T cells. (**H**) UMAP space generated by cell origin and proportion of samples contributing to 4 main T cell clusters in both groups mentioned before. Symbols represent individual data points, with the mean as a bar graph. Data were analyzed using a 2-tailed *t* test. *P < 0.05, **P < 0.01, ***P < 0.001. Scale bars: 25 µm.

To make a detailed assessment of the steroid-induced changes in T cell number and function, we aimed at studying this question in a well-established model of murine cGN (15). In line with protocols used in humans, cGN high-dose steroid treatment (prednisolone 7.5 mg/kg body weight at indicated time points) effectively reduced kidney injury in terms of histopathological and functional kidney damage, and CD3^+^ T cell numbers were significantly decreased in parallel (Figure 2, A and B, and Supplemental Figure 2, A–C). We, furthermore, performed histological, flow cytometric, and single-cell RNA-Seq (scRNA-Seq) analyses after a short high-dose steroid treatment regimen (Figure 2C). In this setting, IHC analyses showed that CD3^+^ T cells were reduced (Figure 2D), as seen in the human data, while numbers of mononuclear phagocytes and neutrophils remained unchanged (Supplemental Figure 3). The CD4^+^ T cell subset was primarily diminished in the kidneys of steroid-treated compared with untreated nephritic mice when further analyzed by flow cytometry, while the CD8^+^ T cell subset was not (Figure 2, E and F). Unsupervised scRNA-Seq analyses of renal CD3^+^ T cells resulted in 9 different clusters in the Uniform Manifold Approximation and Projection (UMAP) space, showing an unexpected paucity of differentially expressed genes (Figure 2G and Supplemental Figure 4, A and B). However, when analyzing the cells by origin — that is, by measuring the respective fraction of sampled cells contributing to specific clusters of interest — we detected a substantial shift in CD4^+^ T cell clusters from memory CD4^+^ T cells toward naive CD4^+^ T cells. By contrast, CD8^+^ T cells and Tregs remained stable (Figure 2H). Analysis of circulating autologous mouse anti-sheep antibodies revealed similar levels of total IgG, as well as IgG subclass antibodies, ruling out a B cell–mediated effect of GC treatment (Supplemental Figure 5).

### CD4 T cell preapoptosis, apoptosis, proliferation, and emigration to the draining renal lymph node (rLN) are unaffected by steroid treatment in murine cGN.

Studies in malignant and in autoinflamatory and inflammatory diseases suggest a significant effect of steroids on leukocyte survival and proliferation (16, 17). Hence, we aimed at analyzing these factors as potential contributors to the reduction in the renal T cell infiltrate found in our model. We performed IHC and flow cytometric analyses of preapoptosis and apoptosis and proliferative capacity of CD4^+^ T cells using the experimental setup described previously (Supplemental Figure 6A). While the evaluation of kidney tissue slides revealed no increase in apoptotic lymphocytes, we found a reduction in proliferating lymphocytes, which is likely to be a consequence of the overall decrease in the T cell infiltrate (Supplemental Figure 6, B and C). Flow cytometric data corroborate these findings by showing unchanged proportions in CD4^+^ T cell preapoptosis and apoptosis and proliferation in the 2 nephritic groups (Supplemental Figure 6D). To rule out an augmented egress of T cells from the kidney through high-dose steroid treatment, we analyzed leukocyte numbers and proportions in the renal draining lymph node, in addition to the renal T cell infiltrate. In steroid-treated mice, the lymph nodes contained significantly fewer total leukocytes, and while the proportion of CD4^+^ T cells of all CD45^+^ leukocytes remained unchanged, their total numbers were significantly reduced, likely following the decreased renal CD4^+^ T cell infiltrate but not causing it (Supplemental Figure 6, E and F).

### GR deficiency in T cells does not prevent the reduction in renal CD4^+^ T cells by GCs.

**Figure 3:**
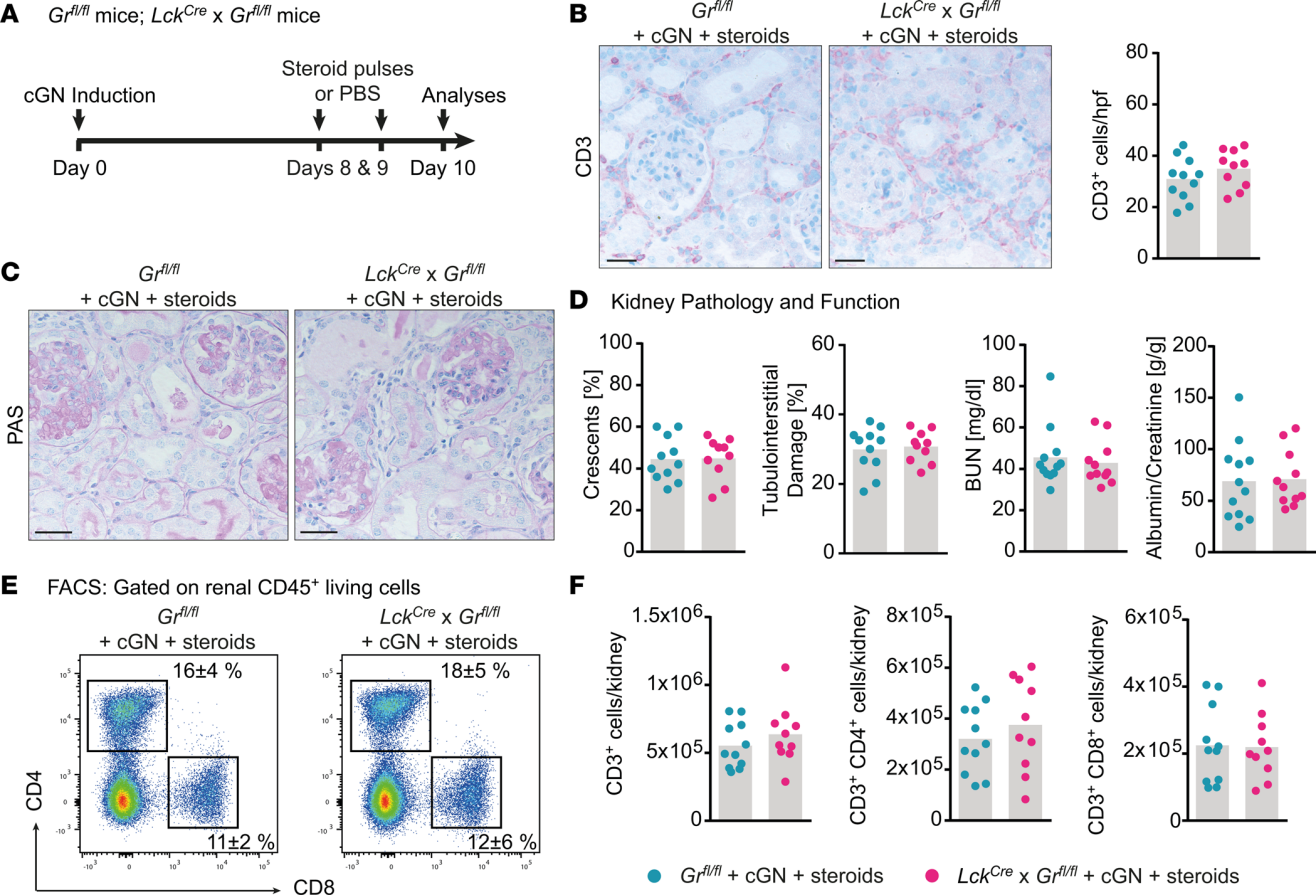
Glucocorticoid receptor (GR) deficiency in T cells does not prevent the reduction in renal CD4^+^ T cells by glucocorticoids. (**A**) Schematic representation of the experimental setup. (**B**) Representative photographs of immunohistochemical CD3 staining and semiquantitative analysis of intrarenal T cell numbers of nephritic *Gr^fl/fl^* and nephritic *Lck^Cre^* × *Gr^fl/fl^* mice with pulsed steroids. (**C**) Representative photographs of PAS-stained kidney sections obtained from the groups mentioned before. (**D**) Quantification of crescent formation, tubulointerstitial damage, BUN levels, and albumin/creatinine ratios determined for the previously mentioned groups. (**E**) Representative plots showing mean percentages of renal CD4^+^ and CD8^+^ T cells. (**F**) Quantification of flow cytometric analyses of changes in CD3^+^, CD3^+^CD8^+^, and CD3^+^CD4^+^ T cells performed for the groups mentioned before. Data are representative of 3 independent experiments. Symbols represent individual data points, with the mean as a bar graph. Data were analyzed using a 2-tailed *t* test. Scale bars: 25 µm.

To test whether the decrease in renal T cell infiltrate by high-dose steroid treatment might be caused by T cell intrinsic factors, we induced cGN in mice deficient in the glucocorticoid receptor (GR), specifically in T cells (*Lck^Cre^* ***×*** *GR^fl/fl^* mice), and in mice without Cre expression using the established steroid treatment approach (Figure 3A). Cre-induced excision of the GR in T cells did not lead to changes in the CD3^+^ T cell infiltrate measured by IHC CD3 staining (Figure 3B) or to differences in kidney damage, in terms of histological and functional parameters (Figure 3, C and D). There was no change in T cell numbers between the 2 groups in flow cytometric analyses of total CD3^+^, CD4^+^, and CD8^+^ T cells (Figure 3, E and F) or cytokine-producing CD4^+^ T cells (Supplemental Figure 7A). The IHC FoxP3 staining performed to quantify Tregs (Supplemental Figure 7B) did not show changes, either. Furthermore, *GR^fl/fl^* mice with and without Cre expression were equally susceptible to cGN induction with comparable parameterse of disease burden (Supplemental Figure 7C). Taken together, these findings argue against direct effects of steroids on T cells in cGN.

### GC treatment changes the renal inflammatory environment to attenuate CD4^+^ T cell recruitment.

**Figure 4:**
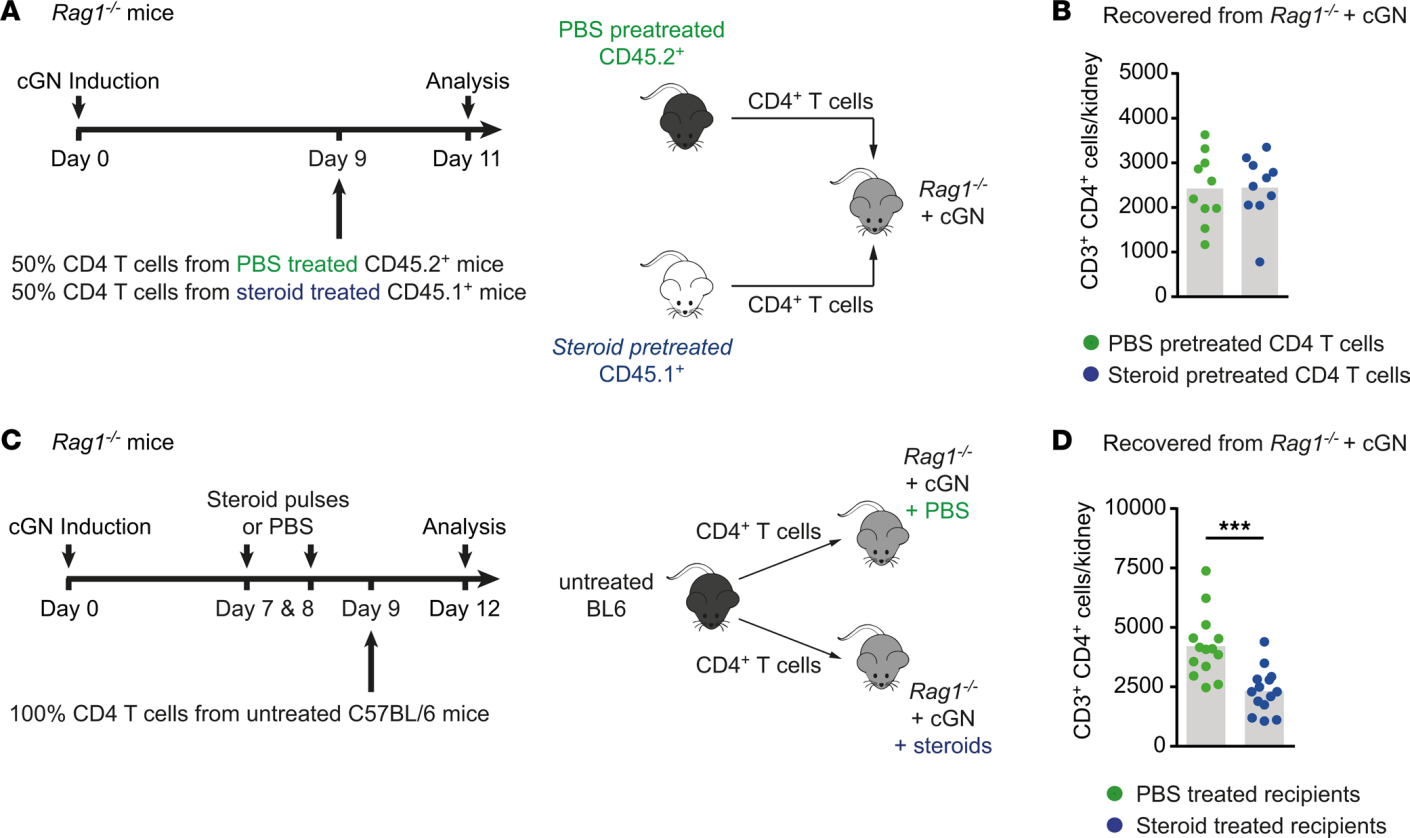
Glucocorticoid treatment changes the renal inflammatory environment to attenuate CD4^+^ T cell recruitment. (**A** and **B**) Schematic representation of the experimental setup, and numbers of recovered renal CD45.1^+^ and CD45.2^+^CD4^+^ T cells from nephritic *Rag1^–/–^* mice after 2 days following i.v. transfer of a mixture of equal numbers of CD45.2^+^CD3^+^CD4^+^ T cells from PBS pretreated mice, and CD45.1^+^CD3^+^CD4^+^ T cells from steroid pretreated mice. (**C** and **D**) Schematic representation of the experimental setup, and numbers of recovered renal CD4^+^ T cells from PBS-treated and steroid-pulsed nephritic *Rag1^–/–^* mice after 3 days following intravenous transfer of CD3^+^CD4^+^ T cells into both groups. Symbols represent individual data points, with the mean as a bar graph. Data were analyzed using a 2-tailed t test. ***P < 0.001.

Next, we sought to investigate whether renal T cell recruitment could be perturbed by steroid treatment — particularly, whether steroids might operate through a direct effect on target cells or whether changes in the inflamed environment could cause the reduction in the renal T cell infiltrate. In a first step, we, therefore, transferred CD4^+^ T cells from nephritic steroid–treated mice or nephritic untreated mice into untreated *Rag1^–/–^* mice (Figure 4A). Flow cytometric analysis showed a comparable ability of CD4^+^ T cells to migrate into the inflamed kidneys of *Rag1^–/–^* recipient mice, regardless of whether these cells had been steroid sensitized or not (Figure 4B).

In a second step, we transferred CD4^+^ T cells from untreated nephritic mice into *Rag1^–/–^* mice previously treated with steroids or into untreated *Rag1^–/–^* mice (Figure 4C). In contrast to the abovementioned result, the analysis of *Rag1^–/–^* recipient mice treated with steroids before the T cell transfer revealed a distinct reduction in renal CD4^+^ T cell numbers compared with untreated *Rag1^–/–^* recipient mice (Figure 4D), arguing for perturbed T cell recruitment through steroid-dependent changes in the renal inflammatory environment.

### The recruitment of CXCR3^+^ Th1 cells in murine cGN is attenuated by GCs through the dampened production of corresponding chemokines by tubular epithelial cells.

**Figure 5:**
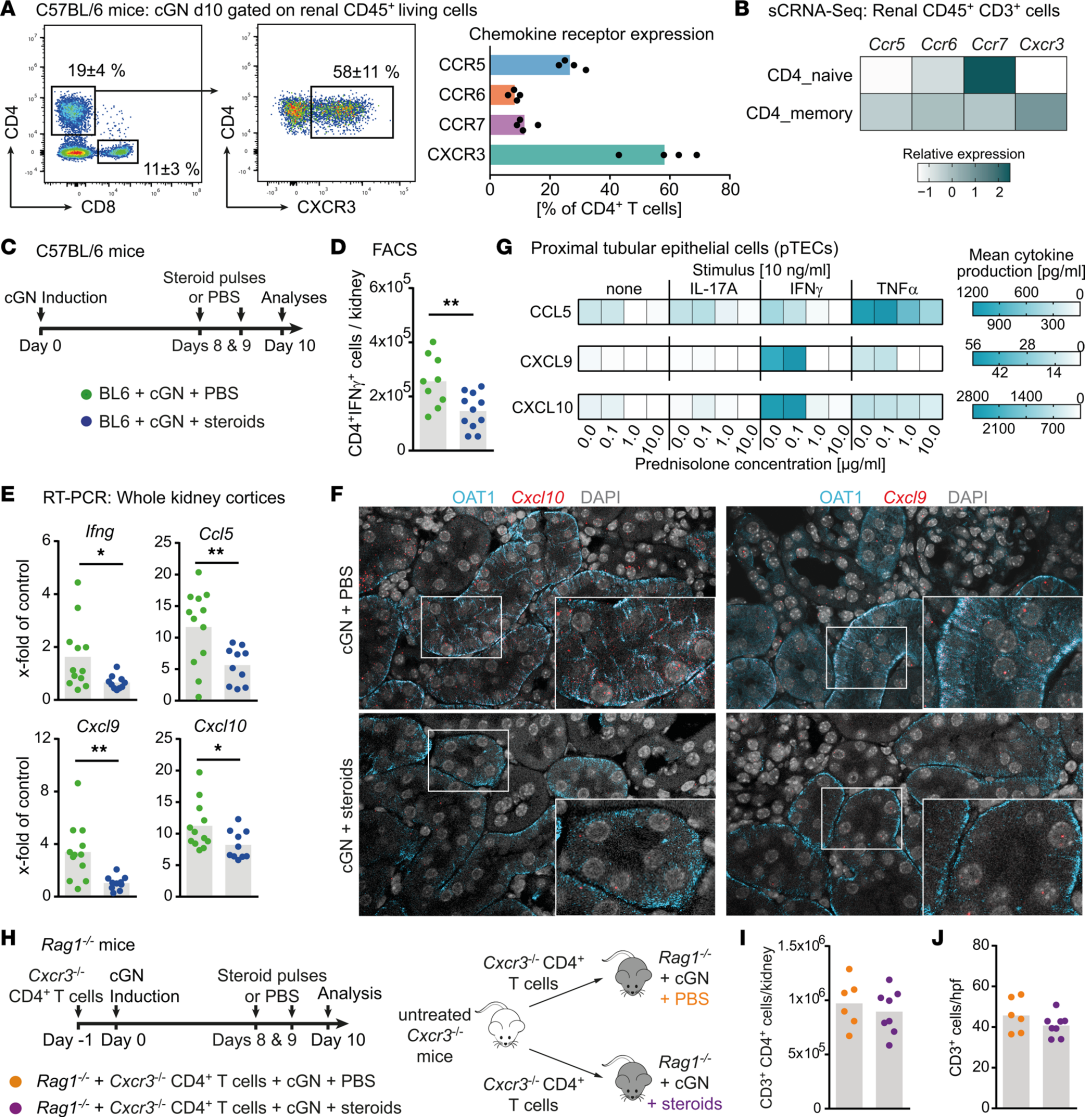
The recruitment of CXCR3^+^ Th1 cells in murine cGN is attenuated by glucocorticoids through the dampened production of the corresponding chemokines by tubular epithelial cells. (**A**) Representative plots showing mean percentages of CD4^+^ and CD8^+^ T cells, respectively, and percentage CXCR3 positivity of CD4^+^ T cells, as well as relative chemokine receptor expression of CD3^+^CD4^+^ T cells obtained from nephritic animals at day 10 of cGN determined by flow cytometric analyses. (**B**) Analysis of the single-cell RNA-Seq data of renal CD3^+^ T cells of nephritic mice analyzed for relative chemokine receptor expression of the CD4_naive and CD4_memory clusters. (**C** and **D**) Schematic representation of the experimental setup, and quantification of flow cytometric analyses of absolute numbers of CD3^+^CD4^+^IFN-γ^+^ T cells isolated from kidneys of untreated and steroid-treated nephritic mice. (**E**) Reverse transcription PCR (RT-PCR) analyses of indicated mRNA expression from whole renal cortices of kidneys collected from the groups mentioned before. (**F**) RNA scope analyses show the preferential localization of *Cxcl9* and *Cxcl10* mRNA to the tubulointerstitial compartment and markedly reduced expression in steroid treated mice. Scale of images is indicated by 1 cm in width equaling 15 µm. (**G**) Heatmap of chemokine protein levels in supernatants of proximal tubular epithelial cells after stimulation with either medium alone, IL-17A, IFN-γ, or TNF-α under increasing concentrations of prednisolone. (**H** and **I**) Schematic representation of the experimental setup, and numbers of recovered renal CD4^+^ T cells from PBS-treated and steroid- pulsed nephritic Rag1^–/–^ mice after eleven days following i.v. transfer of CXCR3^–^CD4^+^ T cells into both groups. (**J**) Semiquantitative analysis of intrarenal T cell numbers derived from kidney sections IHC stained for CD3 for the groups mentioned before. Symbols represent individual data points, with the mean as a bar graph. Data were analyzed using a 2-tailed *t* test. *P < 0.05, **P < 0.01.

After establishing a CD4^+^ T cell recruitment deficit as the primary reason for the reduced T cell infiltrate in kidneys of steroid-treated animals, we assessed the chemokine receptor expression profile of renal CD4^+^ T cells in cGN because the chemokine/chemokine receptor system is the key regulator of directional T cell trafficking under inflammatory conditions (15, 18, 19). On day 10 of cGN, the chemokine receptor CXCR3 was highly expressed on renal CD4^+^ T cells, followed by CCR5, and both are markers of Th1 cells (Figure 5A and Supplemental Figure 8). This result correlates with the finding that the 2 main renal CD4^+^ T cell clusters identified by scRNA-Seq differed in their relative chemokine receptor mRNA expression in a manner allowing the assumption to be made that the predominantly occurring CXCR3-expressing memory CD4^+^ Th1 cells fail to infiltrate the kidney after high-dose steroid treatment (Figure 5B). Likewise, in the experimental setting of a short high-dose steroid treatment regimen (Figure 5C), we found that IFN-γ–producing CD4^+^ T cells were predominantly reduced in kidneys of nephritic steroid–treated mice, while the numbers of IL-17–producing CD4 ^+^ T cells and Tregs were unchanged (Figure 5D and Supplemental Figure 9, A and B). In addition, the corresponding Th1-associated cytokine and chemokine mRNA levels, most importantly *Cxcl9* and *Cxcl10*, were specifically and significantly downregulated after steroid treatment, whereas other targets such as *Il-17a* and *Ccl2* mainly remained unchanged (Figure 5E and Supplemental Figure 9C). Next, we performed in situ hybridization analyses (RNAscope) to localize the renal production of the CXCR3-attracting chemokines CXCL9 and CXCL10, and this production was primarily detectable in cells corresponding to the proximal tubular epithelium in the periglomerular and tubulointerstitial spaces (Figure 5F). In support of this finding, in vitro experiments showed that the coapplication of steroids abolished, in a dose-dependent manner, the cytokine-induced production of CXCL9 and CXCL10 by proximal tubular epithelial cells (pTECs), further corroborating the role of steroids in changing the local proinflammatory environment as a means of regulating the renal T cell infiltrate (Figure 5G and Supplemental Figure 10).

### The CXCL9/CXCL10-CXCR3 axis is a potential target of GCs that provides protection from cGN.

To test whether CXCR3 deficiency abrogates the steroid-dependent reduction in CD4^+^ T cell recruitment to the kidney in cGN, we i.v. transferred *CXCR3^–/–^* CD4^+^ T cells into *Rag1^–/–^* mice. According to the previously used experimental setup, we then treated them with PBS or steroids 2 days before sacrificing them for analyses (Figure 5H). Flow cytometric analyses of renal CD4^+^ T cells revealed comparable numbers of cells in PBS- and steroid-treated animals and similar numbers of CD3^+^ T cells as a result of the quantification of IHC CD3 staining of kidney sections from the groups mentioned above (Figure 5, I and J). No differences were found in kidney damage, as measured by crescent formation (Supplemental Figure 11). These findings additionally underscore the importance of the CXCL9/CXCL10-CXCR3 axis as a significant factor in GC-mediated attenuation of T cell recruitment.

### GC treatment alleviates the recruitment of CXCR3^+^ Th1 cells to the kidneys of patients with ANCA-GN.

**Figure 6:**
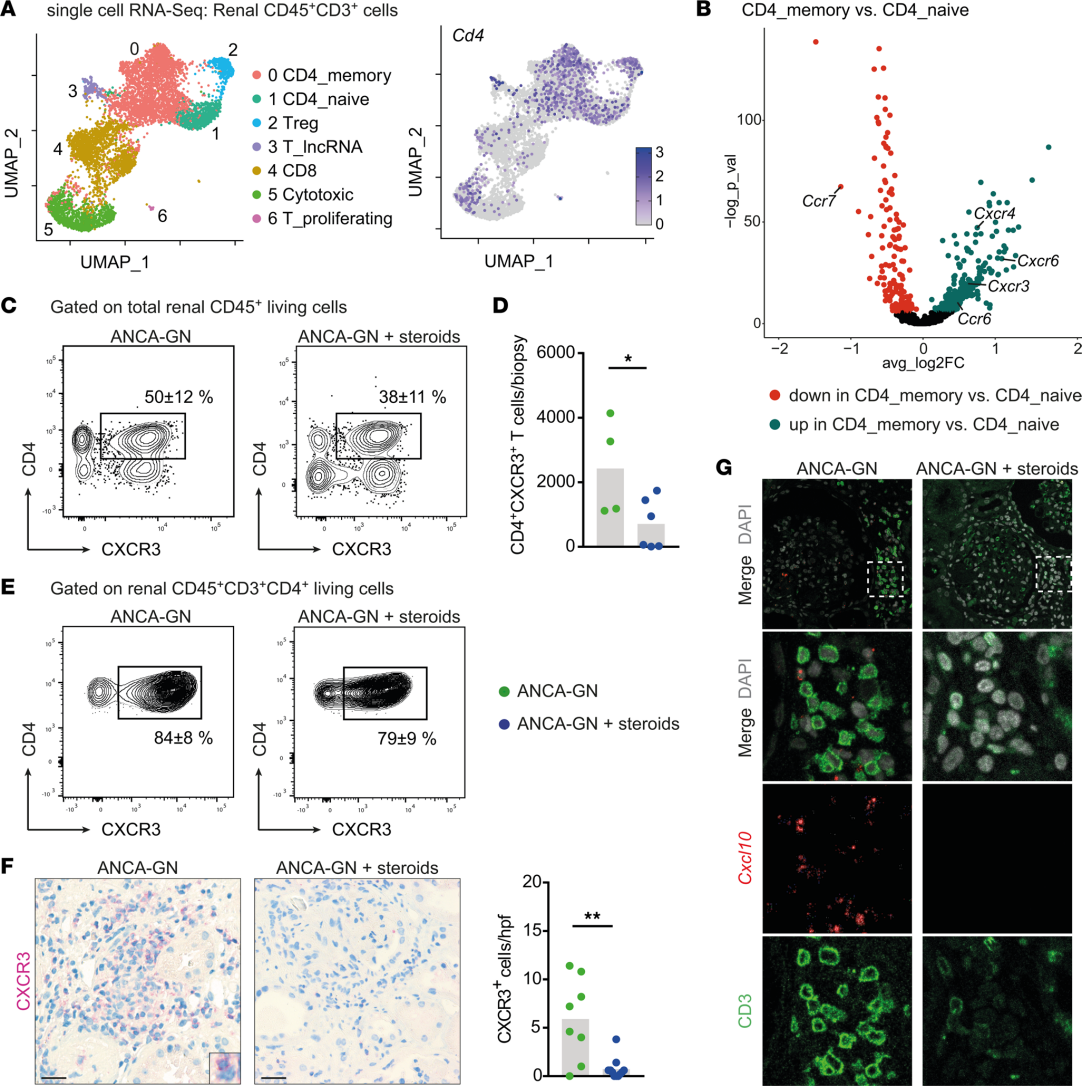
Glucocorticoid treatment alleviates the recruitment of CXCR3^+^ Th1 cells to the kidneys of patients with ANCA-GN. (**A**) UMAP space of 7 clusters defined by unsupervised clustering of renal CD3^+^ T cells from kidney biopsies of patients with ANCA-GN treated with glucocorticoids and UMAP of *Cd4* expression in these clusters. (**B**) Volcano plot of differentially expressed chemokine receptor mRNA of the CD4_memory cluster compared with the CD4_naive cluster. (**C**) Representative flow cytometric plots showing mean percentages of CXCR3 positivity of renal CD45^+^ cells in untreated patients with ANCA-GN and patients with ANCA-GN after glucocorticoid treatment. (**D**) Quantification of total renal CD3^+^CD4^+^CXCR3^+^ T cells in these groups. (**E**) Representative flow cytometric plots showing mean percentages of CXCR3 positivity of renal CD3^+^CD4^+^ T cells in untreated patients with ANCA-GN and patients with ANCA-GN after glucocorticoid treatment. (**F**) Representative photographs of CXCR3-stained kidney sections from kidney biopsies of untreated patients with ANCA-GN and patients with ANCA-GN after glucocorticoid treatment and semiquantitative analysis of intrarenal CXCR3^+^ cells in these groups. (**G**) Combination of immunofluorescence staining of CD3 with *Cxcl10* RNAscope analysis of kidney sections from kidney biopsies of untreated patients with ANCA-GN and patients with ANCA-GN after glucocorticoid treatment. Symbols represent individual data points, with the mean as a bar graph. Scale bar: 25 μm. Data were analyzed using a 2-tailed *t* test. *P < 0.05, **P < 0.01.

scRNA-Seq of CD3^+^ T cells collected from patients with ANCA-GN resulted in 7 clusters in the UMAP space with robust expression of *Cd4* in selected clusters (Figure 6A and Supplemental Figure 12). Analysis of the 2 main CD4^+^ T cell clusters revealed elevated relative expression of CXCR3 — alongside CXCR4, CXCR6, and CCR6 —and a reduced expression of CCR7 in pathogenic CD4^+^ memory T cells relative to naive CD4^+^ T cells (Figure 6B), corresponding to the murine data set shown in Figures 2 and 5. In line with the transcript profiling data, flow cytometric analysis of intrarenal T cells gathered from kidney biopsy material of the same patients revealed significantly reduced absolute CD4^+^CXCR3^+^ T cell numbers in GC-treated patients (Figure 6, C and D). By contrast, the percentage of CXCR3 expression of CD4^+^ T cells was comparable (Figure 6E). This finding was corroborated by IHC CXCR3 staining of kidney sections from patients with ANCA-GN treated with 1 up to 3 pulses of i.v. GCs (ANCA-GN + steroids) or without treatment (ANCA-GN) before kidney biopsy; the staining revealed a reduction in CXCR3^+^ intrarenal cells in the treatment group (Figure 6F). In addition, the combination of immunofluorescence staining of CD3 with *Cxcl10* RNAscope analysis of human kidney sections showed the preferential localization of T cells to periglomerular and tubulointerstitial areas of high *Cxcl10* mRNA expression and markedly reduced *Cxcl10* signals in patients treated with i.v. GCs compared with patients without treatment before kidney biopsy (Figure 6G).

## Discussion

Here, we report that GCs rapidly reduce the numbers of proinflammatory CXCR3^+^CD4^+^ Th1 cells in the kidney and subsequent renal tissue damage in human and experimental crescentic GN. Mechanistically, we demonstrate that the GC-induced decrease in CD4^+^ T cells in the kidney was due to reduced CXCR3^+^ T cell recruitment, as a specific consequence of diminished renal expression of the corresponding chemokines CXCL9 and CXCL10. Our results suggest that the CXCL9/CXCL10-CXCR3 axis is a target of GCs that provides protection against immune-mediated renal injury.

CD4^+^ T cells are key drivers of autoimmune diseases, including crescentic GN. Many of the effects of T cells on renal damage and repair, such as local cytokine production, depend on their presence at the site of inflammation (5). Therefore, our observation that GCs diminished their numbers in the kidney of patients with ANCA-associated GN is of interest; and even if sample sizes were rather small, which increases the risk of sampling bias, we could not detect other major between-group differences. From a conceptual point of view, 4 different factors may determine how GCs can regulate the number of CD4^+^ T cells in the kidney: infiltration of T cells, proliferation of T cells, death/apoptosis of T cells, and egress of T cells out of the kidney. To investigate these different possibilities in an experimental setting, we took advantage of a well-characterized crescentic GN model in mice that is induced by the injection of nephrotoxic serum directed against the glomerular basement membrane (20, 21). This approach triggers a strong T cell response directed against the planted antigen, which results in a CD4^+^ Th1/Th17 cell–driven formation of glomerular crescents, tubulointerstitial injury, and loss of kidney function, resembling some features of human crescentic GN (1).

Our studies show that high-dose GCs significantly reduce the trafficking of CD4^+^ T cells into the inflamed kidney with little short-term influence on other immune cells, incidentally pointing to an underappreciated T cell turnover, but they did not affect T cell death, proliferative capacity, or T cell egress. Our data on T cell death contrast with those gathered from the thymus, where apoptosis induction — as the primary function of endogenous GCs ± is well established (22–24), while our data are in line with the resistance of T cells in the CNS to GC-induced apoptosis and its dispensability for treatment success was found in experimental autoimmune encephalomyelitis (EAE) (25). Moreover, data on the blocking of T cell migration to the CNS in EAE by GC treatment reflect our findings (26), which might lead to a potentially underrated mechanism of GC treatment in different forms of autoimmune inflammatory diseases. Further support for a dominant role of GCs in preventing T cell migration stems from a recent study in a preclinical model of stem cell transplantation, where periprocedural GC administration prevented donor T cell accumulation in the gastrointestinal tract without evidence of influencing apoptosis and proliferation (27).

After identifying diminished recruitment as the main factor regulating the number of CD4^+^ T cells in the kidney following high-dose GC treatment, a T cell–intrinsic GC-mediated effect was ruled out by the finding that T cell–specific deletion of the GR did not reinstall the renal T cell infiltrate. This finding contrasts with data published for EAE. In this model, the involvement of the GR in T cells is vital for the protection from autoimmunity (28).

Mechanistically, we show that in vitro GCs act directly on pTECs, thereby repressing the expression of the CXCR3-corresponding ligands CXCL9 and CXCL10. Furthermore, although in vivo experiments using mice with pTEC-specific deletion of the GR were, so far, not feasible, the combination of immunofluorescence staining and FISH not only revealed pTECs as a major source of CXCL9 and CXCL10, but also showed markedly reduced CXCL9 and CXCL10 expression of pTECs in kidney sections from GC-treated mice. The chemokine receptor CXCR3 is rapidly induced on naive T cells after activation and is highly expressed on Th1 effector CD4^+^ T cells, playing an essential role in T cell trafficking and function (29, 30). Our finding that GCs predominantly acted on resident kidney cells, while the deficiency of the GR in immune cells, in this case specifically in T cells, played no role, corresponds to a recent study showing direct effects of GCs on activated glomerular parietal epithelial cells in experimental cGN (31). Of note, a therapeutic option to modulate parenchymal renal cells (e.g., pTECs) with the aim of generating an antiinflammatory microenvironment in renal autoimmune diseases has recently been proposed (32), and multiple studies underscore the central role of the CXCL9/CXCL10-CXCR3 axis in the development of human and experimental immune-mediated kidney diseases, including ANCA-GN and lupus nephritis, by directing pathogenic effector T cells to the sites of inflammation, thus identifying this pathway as a promising therapeutic target (15, 18, 33–35).

The CXCR3-corresponding ligands CXCL9 and CXCL10 are secreted from cultured tubular cells stimulated with IFN-γ, and their production was inhibited by GCs in a dose-dependent manner, indicating a direct inhibitory effect on intracellular signaling pathways. Therefore, GCs can block the proinflammatory amplification loop of the CXCL9/CXCL10-CXCR3 axis , which is mediated by tissue-invading IFN-γ–producing CXCR3^+^ Th1 cells and the IFN-γ–induced CXCR3 ligands. This may be all the more important, since autoimmune diseases, including autoimmune kidney diseases, constitute a spectrum of disorders that exhibit activity of diverse pathogenic cytokine patterns and involvement of distinct immune cell subtypes. Systemic lupus erythematodes (SLE), for instance, is characterized by a prominent type I IFN signature that correlates with disease activity (36), and a high-dose GC treatment effectively controls disease flares and activity (37, 38). Our results corroborate the notion that high-dose GCs exhibit their diverging potency in autoimmune diseases by preferentially targeting particular prone cytokine pathways and leukocyte subsets (39) — notably, Th1/IFN-γ driven disorders. A recent study identified the expansion of neutrophils, called IFN^active^ neutrophils, with a distinct IFN signaling signature as the main target of beneficial GC treatment effects in patients with COVID-19, and this expansion may also stem from GCs selectively targeting the Th1/IFN-γ pathogenic cytokine pathway (40).

In conclusion, we identified the CXCL9/10-CXCR3 pathway as a previously unknown specific mechanism by which GCs promote their therapeutic effects in experimental and human cGN. A better understanding of the precise targets of GCs based on their modes of action is fundamental to the development of new treatment strategies with fewer severe side effects in autoimmune and chronic inflammatory diseases.

## Methods

### Animal experiments.

*GR^fl/fl^* mice and *Lck^Cre^* × *GR^fl/fl^* mice were provided by M. Friese (ZMNH). CD45.1 mice and *Rag1^–/–^* mice were purchased from The Jackson Laboratory. *Cxcr3*^–/–^ mice were generated by our group and bred at the animal facility of the University Medical Center Hamburg-Eppendorf. All mice were on the C57BL/6J background, and KO mice underwent embryo transfer to meet the general standards of our institution. Age-matched C57BL/6J WT controls were also bred in our animal facility, and all animals were raised in specific pathogen-free conditions. In intervention experiments, the mice received either 7.5 mg/kg body weight prednisolone (MIBE GmbH Arzneimittel) in 150 μL sterile PBS at room temperature (dosage based on those used in patients with ANCA-GN), or just 150 μL sterile PBS at room temperature via the lateral tail vein for 2 or 7 consecutive days, depending on the experimental setup.

### Induction of experimental GN and functional studies.

Nephrotoxic nephritis was induced by i.p. injection of 2.5 mg nephrotoxic sheep serum per gram of body weight into 8- to 12-week-old male mice as previously described (15). For urine sample collection, mice were housed in metabolic cages for 5 hours. Urinary albumin excretion was determined by standard ELISA analysis (Mice-Albumin Kit, Bethyl Laboratories), while urinary creatinine, BUN, and serum creatinine were measured using standard laboratory methods.

### Quantitative PCR (qPCR) analyses.

Total RNA from the renal cortex was isolated with the NucleoSpin Kit (Macharey-Nagel) in accordance with the manufacturer’s protocol. RNA was reverse transcribed with the High-Capacity cDNA Reverse Transcription Kit (Thermo Fisher Scientific). qPCR of different chemokines was performed using specific primers, and IFN-γ was determined with TaqMan PCR (Mm01168134, Invitrogen). Measurement was performed on a StepOnePlus Real-Time PCR system (Thermo Fisher Scientific) as previously described, with the 18S rRNA as a housekeeping gene and all samples run in duplicate.

### Morphological analyses of murine and human tissues.

In murine tissue, glomerular injury and crescent formation, deposition of PAS^+^ material, and tubulointerstitial injury were assessed as described (41). In brief, paraffin-embedded sections (2 μm) were stained with antibodies directed against CD3 (A0452, Dako), FoxP3 (FJK-16s, eBioscience), F4/80 (BM8; Dianova BMA), Gr-1 (NIMP-R14; Hycult Biotech, Uden), Ki67 (D3B5, Cell Signaling), cleaved caspase 3 (cC3) (Asp175; Cell Signaling Technology), and OAT1 (OAT11-A; Alpha Diagnostics International). Tubulointerstitial CD3^+^ cells in 30 high-power fields (total original magnification, ×400) per kidney, tubulointerstitial FoxP3^+^ cells in 30 high-power fields (total original magnification, ×400) per kidney, tubulointerstitial F4/80^+^ cells in 30 high-power fields (total original magnification, ×400), tubulointerstitial GR-1^+^ cells in 20 low-power fields (total original magnification, ×200), tubulointerstitial Ki-67^+^ cells in 30 high-power fields (total original magnification, ×400) per kidney, and tubulointerstitial cC3^+^ cells in 30 high-power fields (total original magnification, ×400) per kidney were counted in a blinded manner. In human kidney tissue, the histopathologic kidney injury was assessed by a nephropathologist according to standardized algorithms, and the Renal Risk Score was assessed as described (14). For IHC analysis, human paraffin-embedded kidney sections (2 μm) from renal biopsies obtained from patients with ANCA-GN were stained with an antibody directed against CD3 (A0452, Dako, Glostrup) and CXCR3 (1C6; BD Biosciences). Renal CD3^+^ and CXCR3^+^ cells in 20 high-power fields per renal biopsy (total original magnification, ×400) were counted. For immunofluorescence staining of CD3, human paraffin-embedded kidney sections (2 μm) from patients with ANCA-GN were stained with an antibody directed against CD3 (DAKO, A0452) after dewaxing and antigen retrieval (pH 6 for 15 minutes). All slides were evaluated using an Axioskop light microscope (Zeiss) and photographed with an Axiocam HRc (Zeiss) or by confocal microscopy with an LSM800 meta microscope (Zeiss) using the LSM software (Zeiss).

### RNAscope (mRNA FISH).

*Cxcl9* and *Cxcl10* mRNA detection in mice kidney sections and *Cxcl10* mRNA detection in human kidney sections were manually carried out using the RNAscope Multiplex Fluorescent Assay (Advanced Cell Diagnostics) according to the manufacturer’s instructions. Briefly, 2–4 μm formalin-fixed, paraffin-embedded (FFPE) slides were baked at 60°C for 1 hour, deparaffinized twice in xylene for 5 minutes each time, and incubated twice in 100% ethanol for 2 minutes each time. The sections were then air dried and treated with RNAscope hydrogen peroxide solution (catalog 322381, Advanced Cell Diagnostics) for 10 minutes at room temperature and washed with distilled water. Subsequently, they were incubated with target retrieval reagent (catalog 322000, Advanced Cell Diagnostics) at a boiling temperature using BRAUN food steamer (FS3000, BRAUN) for 15 minutes and then washed with distilled water. A hydrophobic barrier was drawn around the sections using an ImmEdge Hydrophobic Barrier Pen (catalog H-4000, Vector Laboratories). The sections were then treated with protease plus reagents at 40°C (catalog 322381, Advanced Cell Diagnostics) for 30 minutes and incubated at 40°C for 2 hours using probes mixed with Mm-Cxcl9-C2 probe (489341-C2, Advanced Cell Diagnostics) and Cxcl10-C3 probe (408921-C3, Advanced Cell Diagnostics) or Hs-CXCL10-C2 (311851-C2, Advanced Cell Diagnostics) diluted at a 50:1 ratio. The slides were repeatedly washed with wash buffer reagent (catalog 310091, Advanced Cell Diagnostics) after each amplification step by RNAscope Multiplex Fluorescent Detection Reagent (catalog 323110, Advanced Cell Diagnostics). All slides were imaged using a Zeiss LSM800 confocal microscope.

### Cell isolation, stimulation, and transfer in mice.

Previously described methods for leukocyte isolation from murine kidneys and spleens were used (41). Cell viability was assessed by trypan blue staining before stimulation, flow cytometry, and cell transfer experiments. For stimulation, after generation of a single-cell suspension as mentioned above, isolated renal leukocytes were activated by incubation at 37°C and 5% CO_2_ for 4.5 hours with PMA (5 ng/mL; MilliporeSigma) and ionomycin (1 μg/mL; Merck Millipore) in RPMI 1640 (Thermo Fisher Scientific) with 10% FCS. After 30 minutes of incubation, Brefeldin A (10 μg/mL; MilliporeSigma) was added. For CD4^+^ T cell transfer experiments, CD4^+^ T cells were isolated from the respective C57BL/6J WT, CD45.2, and *Cxcr3^–/–^* mice using a magnetic cell separation (MACS) CD4^+^ T cell isolation kit (Miltenyi Biotec) according to the manufacturer’s instruction. Viable cells were counted using trypan blue staining, and 2 × 10^6^ live cells were i.v. injected into *Rag1^–/–^* mice 9 days after or 1 day before induction of cGN.

### Human studies including isolation and flow cytometric analyses of human leukocytes.

Kidney biopsies for immunopathologic analyses, flow cytometry, and scRNA-Seq were obtained according to standardized operating procedures from patients with (suspected) ANCA-associated glomerulonephritis and subsequent morphological and clinical parameters were analyzed after inclusion into the Hamburg Glomerulonephritis Registry. Kidney biopsy cores meant for obtaining single-cell suspensions were instantly processed by enzymatic digestion in RPMI 1640 medium with collagenase D at 0.4 mg/mL (Roche) and DNase I (10 μg/mL, Sigma-Aldrich) at 37°C for 30 minutes, followed by dissociation with gentleMACS (Miltenyi Biotec). Samples were filtered through a 30 μm filter (Partec) before antibody staining and flow cytometry. In each case, every single-cell suspension generated from individual kidney biopsy cores was measured in its entirety.

### Flow cytometry and FACS.

Measurements were performed on a BD FACS LSR II (BD Biosciences), while FACS was performed on a FACS AriaFusion or AriaIIIu (BD Biosciences). The data were analyzed using the FlowJo software (Tree Star Inc.). To minimize unspecific antibody binding, cells were either incubated with normal mouse serum (Thermo Fisher Scientific) or with Human BD FC Block (BD Biosciences) before staining for 10 minutes. Murine cells were stained with fluorochrome-labeled antibodies against CD45 (30-F11; BioLegend), CD3 (eBio500A2; eBioscience), CD4 (RM4-5; BioLegend), CD8 (53-6.7; BioLegend), CCR5 (HM-CCR5; BioLegend), CCR6 (29-2L17; BioLegend), CCR7 (4B12; BioLegend), CXCR3 (S18001A; BioLegend), γδTCR (UC7-13D5; BioLegend), NK1.1 (PK136; eBioscience), IL-17A (TC11-18H10.1; BioLegend), IFN-γ (XMG1.2; BD), CD11b (M1/70; eBioscience), F4/80 (BM8; eBioscience), and Ly6G (1A8; R&D Systems); human cells were stained with fluorochrome-labeled antibodies against CD45 (HI30; BioLegend), CD3 (OKT3; BioLegend), CD4 (RPA-T4; BioLegend), CD8 (RPA-T8; BD), CXCR3 (G025H7; BioLegend), and γδTCR (B1.1; eBioscience), as previously described (42). LIVE/DEAD staining (Near-infrared, Invitrogen Molecular Probes) was used to exclude dead cells during flow cytometry and to ensure cell viability after the stimulation procedure. For intracellular staining, samples were processed using Cytofix/Cytoperm (BD Biosciences) according to the manufacturer’s instructions.

### scRNA-Seq of renal CD3^+^ T cells.

scRNA-Seq was performed using the 10X Chromium Controller (10X Genomics), and single-cell libraries were generated with the 10X Genomics Chromium Single Cell 5′v1.1 reagents kit according to the manufacturer’s instructions. cDNA (50 nm) was used for gene expression library construction. Quality control (QC) was performed with hsDNA Qubit (Thermo Fisher Scientific) and BioAnalyzer (Agilent). The libraries were sequenced on an Illumina NovaSeq 6000 system (S4 flow cell) with 150 bp and paired-end configurations.

### Preprocessing and QC of scRNA-Seq data.

10X Genomics raw sequencing data were processed using CellRanger software (version 3.0.2, 10X Genomics). For the mouse data, the 10X Genomics mouse genome mm10 (3.0.0 release) was used as the reference genome (function cellranger count). For the patient data, the human reference genome hg19 (1.2.0 release) was used. The matrices of cells and the unique molecular identifier (UMI) count were obtained and further processed with the R package Seurat (version 4.0.4) (43). For QC of the mouse data, we first filtered out cells in which fewer than 200 genes were detectable. To remove potential doublets, cells with more than 5,000 expressed genes (nFeature) were excluded. We removed low-quality cells with more than 2.5% mitochondrial genes among all detected genes. For the patient data, the overall number of genes detected in each cell was less than the mouse data. We kept the cells with 200–3,000 genes detectable and with less than 10% mitochondrial genes among all genes.

### Dimensionality reduction and clustering.

The Seurat R package (version 4.0.4) was used to perform unsupervised clustering analysis on scRNA-Seq data. Gene counts for cells that passed QC were normalized by library size and log-transformed (function NormalizeData, normalization.method = “LogNormalize”, scale.factor = 10,000). Then, highly variable genes were detected (function FindVariableFeatures, selection.method = “vst”, nfeatures = 2,000). To reduce batch effects, we applied the “anchor” integration method (functions FindIntegrationAnchors and IntegrateData, dims = 1:30) (44). The integrated matrix was then scaled with the ScaleData function (default parameters). PCA was performed on the scaled data (function RunPCA) to reduce dimensionality. Thirty principal components were selected for clustering based on the elbow of a PCA scree plot . The selected principal components were then used to compute the KNN graph based on the Euclidean distance (function FindNeighbors). Cell clusters were subsequently generated using the function FindClusters. The resolution parameters of the FindClusters function for the mouse and human data set were set to be 0.15 and 0.3, respectively, determined by exploration of the top marker genes of each cluster. UMAP was used to visualize the clustering results. The top differentially expressed genes in each cluster were found using the FindAllMarkers function (min.pct = 0.25, logfc.threshold = 0.5) with Wilcoxon rank-sum tests. The most highly expressed genes were then used to determine the cell type of each cluster. The clusters with low CD3 gene counts were removed for downstream interpretation.

### Differential gene expression analysis.

The Seurat FindMarker function (Wilcoxon rank-sum test) was used to perform differential gene expression analysis for each cell type between the control and steroid groups in the mouse data set.

### Culture and stimulation of mouse kidney tubular cells.

Mouse kidney tubular cells (45) were cultured in DMEM (Invitrogen) containing 3%–10% FCS (Thermo Fisher Scientific), 100 U/mL penicillin, and 100 μg/mL streptomycin (Invitrogen) at 37°C with 5% CO_2_. Before stimulation, confluent cells were incubated in serum-free DMEM for 24 hours. Cells were stimulated with either 10 ng/mL of IL-17A, IFN-γ, or TNF-α (all from PeproTech) and various concentrations of prednisolone (MIBE GmbH Arzneimittel). Chemokine levels in supernatants were determined after 24 hours of incubation by using bead-based immunoassay technology.

### Chemokine measurement.

We used a bead-based immunoassay technology (LEGENDplex, BioLegend) to quantify the concentration of cytokines in supernatants of mouse kidney tubular cells. The premixed Mouse Proinflammatory Chemokine panel (catalog 740370) was applied to analyze the relevant chemokines by following the manufacturer’s protocol. Values below the limit of detection were considered zero.

### Assessment of the antigen-specific humoral immune response.

Mouse anti-sheep IgG antibody titers were measured by ELISA using sera collected 10 days after induction of nephritis. In brief, ELISA microtiter plates were coated with 100 μL sheep IgG (100 μg/mL; MilliporeSigma) in carbonate–bicarbonate buffer overnight at 4°C. After blocking with 1% BSA in Tris-buffered saline (MilliporeSigma), plates were incubated with serial dilutions of mouse serum for 1 hour at room temperature. Bound mouse IgG was detected using peroxidase-conjugated goat anti–mouse IgG (HAF007, R&D Systems), TMB peroxidase substrate, and absorbance readings (450 nm). Lack of cross-reactivity of the secondary antibody with sheep IgG was demonstrated by omitting the primary antibody. For the detection of IgG isotypes, the following antibodies have been used: IgG1 (SBA-1070-05; Southern Biotech), IgG2a/2c (610220; Invitrogen), IgG2b (610320; Invitrogen), and IgG3 (115-035-20; Jackson Immuno Research).

### Data and materials availability.

The raw and processed sequencing data are deposited at Gene Expression Omnibus (GEO) repository under accession no. GSE217508.

### Statistics.

The results are shown as the mean presented as a bar graph and superimposed single data points in a scatter dot plot. Differences between 2 individual experimental groups were compared using a 2-tailed *t* test. In the case of 3 or more groups, a 2-way ANOVA with Bonferroni’s multiple-comparison test was used. Experiments that did not yield enough independent data for statistical analysis because of the experimental setup were repeated at least 3 times. *P* < 0.05 was considered as statistically significant.

### Study approval.

All human studies were approved by the local ethics committee of the chamber of physicians in Hamburg (Ethik-Kommission der Ärztekammer Hamburg, registration numbers PV5026 and PV4806) and the Collaborative Research Centre (CRC) Board of the Hamburg Glomerulonephritis Registry (Central Service Project C1 of CRC1192). Written informed consent was obtained from all patients prior to their participation, and all studies were conducted in accordance with the ethical principles stated by the Declaration of Helsinki. All animal experiments were performed according to national and institutional animal care and ethical guidelines and were approved by Behörde für Gesundheit und Verbraucherschutz Hamburg.

## Author contributions

Conceptualization was contributed by JHR and UP; acquisition, analysis, and interpretation of data were contributed by JHR, LR, HJP, YZ, NA, NS, AP, AK, AB, GT, LS, NMT, EH, UOW, TBH, TW, JET, CFK, and UP; writing was contributed by JHR and UP; visualization was contributed by JHR; and supervision was contributed by UP. All authors approved the final version of the manuscript.

## Supplementary Material

Supplemental data

## Version 1 11/10/2022

In-Press Preview

## Version 2 01/10/2023

Electronic publication
