# Supplementary material for: Glucocorticoids target the CXCL9/CXCL10-CXCR3 axis and confer protection against immune-mediated kidney injury
Source: JCI Insight. 2023 Jan 10;8(1):e160251. doi: 10.1172/jci.insight.160251 (PMC9870076; doi:10.1172/jci.insight.160251)
Supplement: Supplemental data [file jciinsight-8-160251-s230.pdf]

	Number	Sex (f / m)	Mean age (years)	ANCA antigen (PR3 / MPO)	Mean Creatinine at the time of Bx (mg/dl)	Mean ACR at the time of Bx (g/g)	Mean Renal Risk Score (0-11)	Numbers of: Histology/ FACS/ scRNA seq
ANCA-GN	9	3 / 6	65	2 / 7	3.3	0.79	4.8	9 / 4 / 0
ANCA-GN + steroids	11	4 / 7	67	3 / 8	3.9	0.82	5.0	11 / 6 / 6

Supplemental Figure 1. Basic clinical characteristics of ANCA-GN patients included in the analyses. Patients either untreated or treated with intravenous glucocorticoids at the time of kidney biopsy included in histological, flow cytometric, and scRNA sequencing analyses.

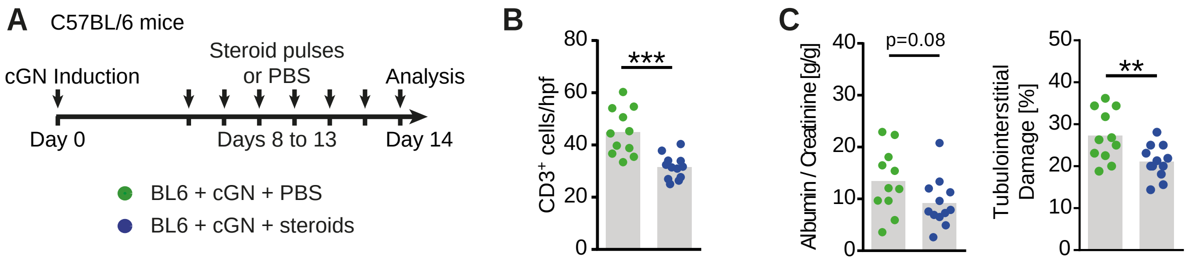

Supplemental Figure 2. Prolonged steroid treatment reduces the CD3 T-cell infiltrate and attenuates murine cGN. (**A**) Schematic representation of the experimental setup. (**B**) Semiquantitative analysis of intrarenal T-cell numbers derived from immunohistochemical CD3 staining of untreated nephritic mice and mice treated for six consecutive days with high doses of steroids. (**C**) Albumin/creatinine ratios and tubulointerstitial damage score determined for the groups mentioned before. Data are representative of three independent experiments. Symbols represent individual data points, with the mean as a bar graph. $^{**}p<0.01$, $^{***}p < 0.001$.

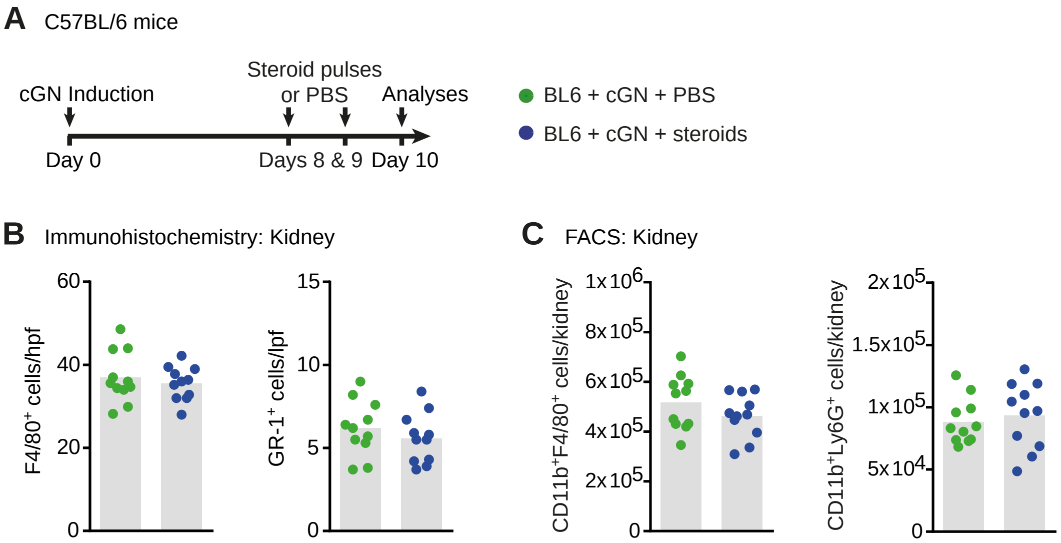

Supplemental Figure 3. Mononuclear phagocytes and neutrophils are no immediate target of GCs in murine cGN

(**A**) Schematic representation of the experimental setup. (**B**) Quantification of immunohistochemical F4/80 as well as GR-1 staining of kidney sections in untreated and steroid-treated nephritic mice. (**C**) Quantification of flow cytometric analyses of renal CD11b^{+}F4/80^{+} as well as CD11b^{+}Ly6G^{+} cells in the previously mentioned groups. Data were analyzed using a two-tailed t test.

A Cluster defining genes

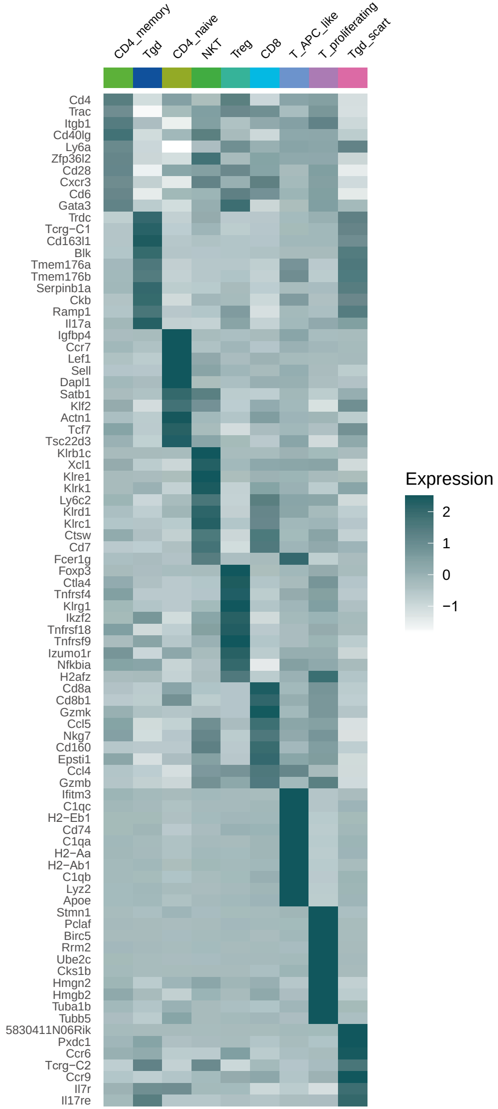

B Differentially expressed genes

p value	logFC	pct.1	pct.2	adjusted p value	gene	cluster	compared to
2.808543e-24	2.4837233	0.111	0.004	4.426545e-20	Iglc1	0	0_Steroid_Control
2.174787e-26	0.4535729	0.822	0.661	3.427682e-22	Lyz2	0	0_Steroid_Control
3.297244e-19	0.4178178	0.786	0.645	5.196786e-15	Apoe	0	0_Steroid_Control
3.044907e-11	0.2975051	0.853	0.782	4.799078e-07	H2-Ab1	0	0_Steroid_Control
3.781174e-07	0.2679631	0.226	0.128	5.959509e-03	Zdhhc18	0	0_Steroid_Control
6.371519e-08	0.2555112	0.195	0.097	1.004215e-03	Cdk2ap1	0	0_Steroid_Control
4.474471e-07	-0.2892632	0.665	0.772	7.052213e-03	Zfp36l2	0	0_Steroid_Control
1.895420e-18	0.5221452	0.849	0.701	2.987372e-14	Lyz2	1	1_Steroid_Control
6.024335e-17	0.4741725	0.842	0.642	9.494955e-13	Apoe	1	1_Steroid_Control
5.186216e-07	0.4671965	0.412	0.246	8.173995e-03	Cebpb	1	1_Steroid_Control
1.774453e-06	0.3716512	0.525	0.402	2.796716e-02	Ppp1r14b	1	1_Steroid_Control
1.614347e-06	0.3020240	0.664	0.551	2.544373e-02	Fgl2	1	1_Steroid_Control
2.699107e-10	0.2989487	0.896	0.827	4.254063e-06	H2-Ab1	1	1_Steroid_Control
3.167964e-06	0.2660604	0.800	0.751	4.993028e-02	Dnaja1	1	1_Steroid_Control
1.680363e-09	-0.2811829	0.955	0.974	2.648420e-05	Ltb	1	1_Steroid_Control
4.303485e-07	-0.2909225	0.904	0.974	6.782723e-03	Cd82	1	1_Steroid_Control
4.630202e-12	-0.3422556	0.748	0.880	7.297661e-08	Cox7a2l	1	1_Steroid_Control
6.669190e-07	-0.3448818	0.153	0.299	1.051131e-02	Sdhaf1	1	1_Steroid_Control
3.593588e-07	-0.4016716	0.268	0.446	5.663855e-03	Asb2	1	1_Steroid_Control
8.724959e-17	0.6225824	0.755	0.527	1.375141e-12	Apoe	2	2_Steroid_Control
4.295069e-12	0.4506000	0.793	0.639	6.769459e-08	Lyz2	2	2_Steroid_Control
1.694570e-08	0.3211479	0.842	0.739	2.670812e-04	H2-Ab1	2	2_Steroid_Control
8.846355e-12	0.2797942	0.995	0.988	1.394274e-07	Gm11808	2	2_Steroid_Control
1.581524e-07	0.6672241	0.449	0.191	2.492640e-03	Ckb	3	3_Steroid_Control
6.574553e-08	0.5029608	0.850	0.552	1.036215e-03	Apoe	3	3_Steroid_Control
2.353900e-07	0.4797140	0.853	0.715	3.709982e-03	Apoe	4	4_Steroid_Control
1.226170e-06	0.3660309	0.922	0.830	1.932567e-02	H2-Ab1	4	4_Steroid_Control
8.421392e-07	0.2635683	0.116	0.000	1.327296e-02	Iglc1	4	4_Steroid_Control
2.118438e-07	0.8514576	0.577	0.277	3.338870e-03	Gzmb	5	5_Steroid_Control
3.093018e-07	0.3244603	0.144	0.000	4.874906e-03	Iglc1	5	5_Steroid_Control
1.860976e-07	0.2873754	0.181	0.000	2.933085e-03	Iglc1	6	6_Steroid_Control
2.164085e-06	0.2554278	0.953	0.928	3.410814e-02	Uba52	6	6_Steroid_Control
1.843690e-06	-0.3946390	0.016	0.203	2.905840e-02	Igkc	6	6_Steroid_Control
2.661965e-10	0.5340029	0.950	0.844	4.195523e-06	Apoe	7	7_Steroid_Control
1.423834e-09	0.4639441	0.962	0.892	2.244105e-05	Lyz2	7	7_Steroid_Control

Supplemental Figure 4. Cluster-defining genes and differentially expressed genes.

(**A**) Cluster-defining genes of nine clusters gathered from unsupervised analysis of single-cell RNA sequencing of renal $CD3^+$ T cells from nephritic C57BL/6 mice with and without steroid treatment at day 10 of cGN. (**B**) List of differentially expressed genes (DEG) in $CD3^+$ T cells from nephritic BL6 mice at day 10 of cGN compared to T cells from steroid-treated mice.

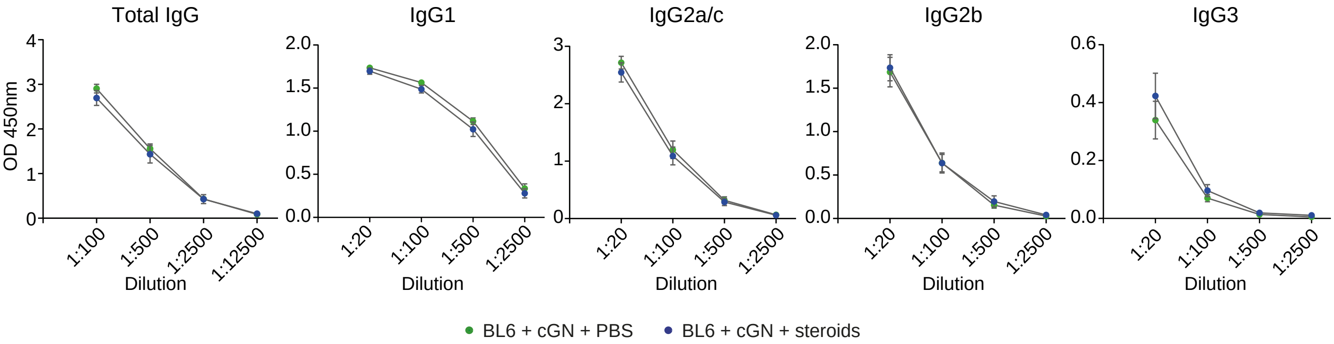

Supplemental Figure 5. GC treatment has no influence on the autologous humoral immune response in murine cGN.

ELISA analyses of circulating serum mouse anti-sheep total IgG-, IgG1-, IgG2a/2c-, IgG2b-, and IgG3-levels at different dilutions from nephritic C57BL/6 mice with (n=16) and without (n=15) steroid treatment at day 10 of cGN. Symbols represent the mean with SEM.

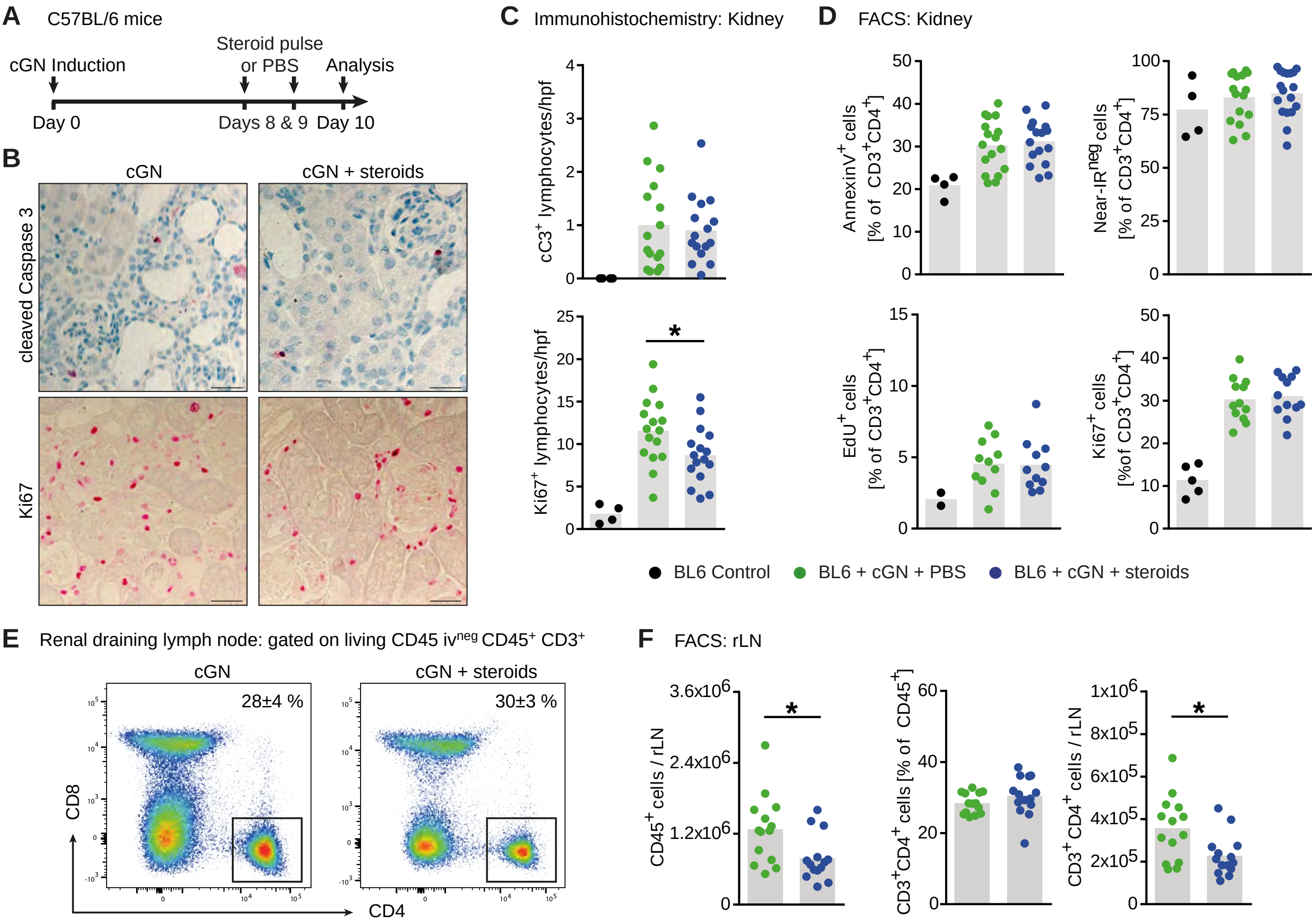

Supplemental Figure 6. CD4 T-cell (pre-)apoptosis, CD4 T-cell proliferation, and CD4 T-cell emigration to the draining renal lymph node (rLN) are unaffected by steroid treatment in murine cGN.

(**A**) Schematic representation of the experimental setup. (**B** and **C**) Representative photographs and quantification of cleaved caspase 3- (cC3) and Ki67-stained kidney sections from healthy mice and nephritic mice with and without steroid treatment. (**D**) Quantification of flow cytometric analyses of (pre-)apoptosis markers AnnexinV and Near-Infrared (Near-IR), as well as proliferation markers 5-ethynyl-2'-deoxyuridine (EdU) and Ki67, on $CD3^+CD4^+$ T cells isolated from kidneys of the previously mentioned groups. (**E**) Representative plots and (**F**) quantification of flow cytometric analyses of absolute numbers and percentages of $CD45^+$ total leukocytes and $CD3^+CD4^+$ T cells isolated from renal lymph nodes of nephritic mice with and without steroid treatment. Data are representative of three independent experiments. Scale bar: 25µm. Symbols represent individual data points, with the mean as a bar graph. Data were analyzed using a two-tailed t test. *p<0.05.

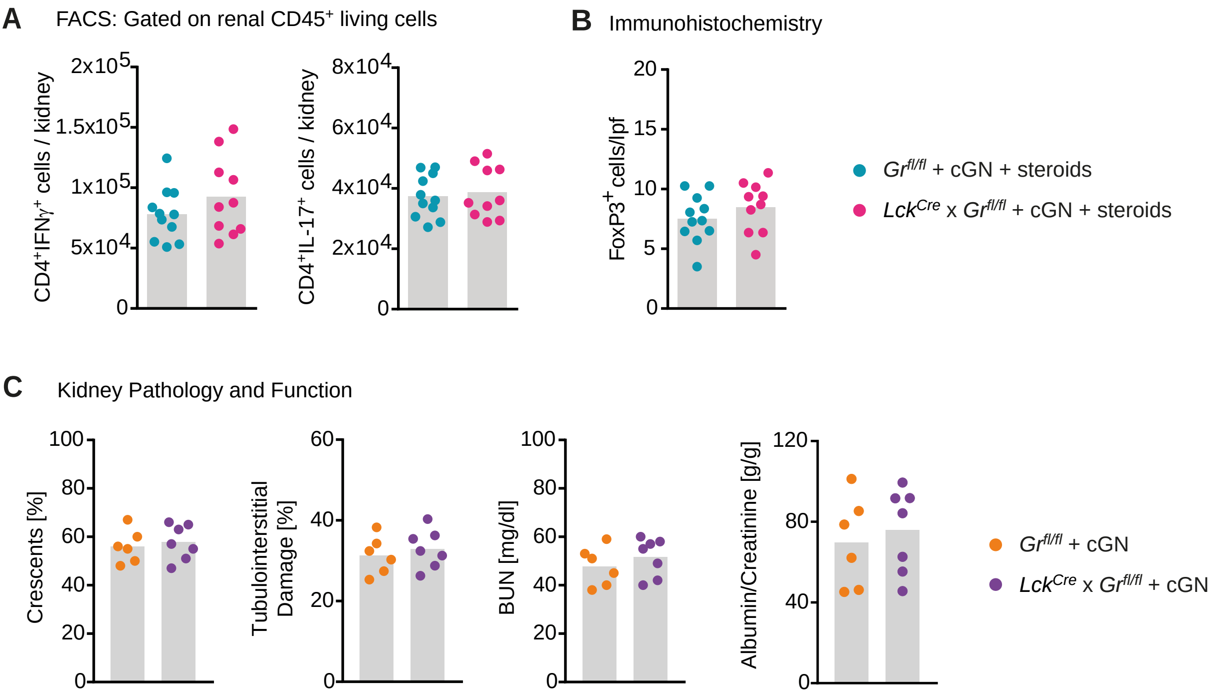

Supplemental Figure 7. T-cell specific deletion of the glucocorticoid receptor (GR) has no significant influence on renal IFNy-, IL-17A-producing CD4$^+$ T cells, or regulatory T cells upon steroid treatment in murine cGN.
(**A**) Quantification of flow cytometric analyses of cytokine-producing CD4$^+$ T cells of nephritic *Gr$^{fl/fl}$* and *Lck^{Cre}* x *Gr$^{fl/fl}$* mice with steroid treatment. (**B**) Quantification of immunohistochemical FoxP3 staining performed for the previously mentioned groups. Data are representative of three independent experiments. (**C**) Quantification of crescent formation, tubulointerstitial damage, BUN levels, and albumin/creatinine ratios in nephritic *Gr$^{fl/fl}$* and *Lck^{Cre}* x *Gr$^{fl/fl}$* mice. Data were analyzed using a two-tailed t test.

C57BL/6 mice: cGN d10 gated on renal CD45$^+$ CD4$^+$ living cells

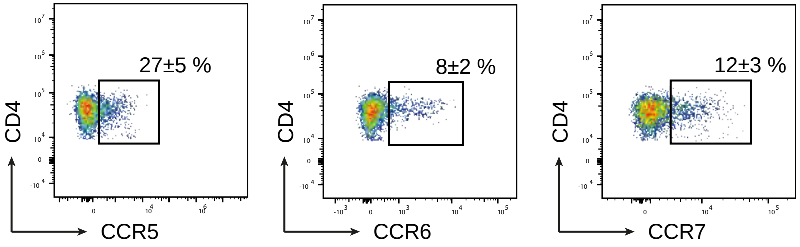

Supplemental Figure 8. Relative expression of selected chemokine receptors of CD4$^+$ T cells on day 10 of murine cGN.

Representative flow cytometric plots show the percentage of CCR5, CCR6, and CCR7 positivity of CD4$^+$ T cells and of CD3$^+$CD4$^+$FoxP3^{neg} T cells from nephritic animals at day 10 of cGN.

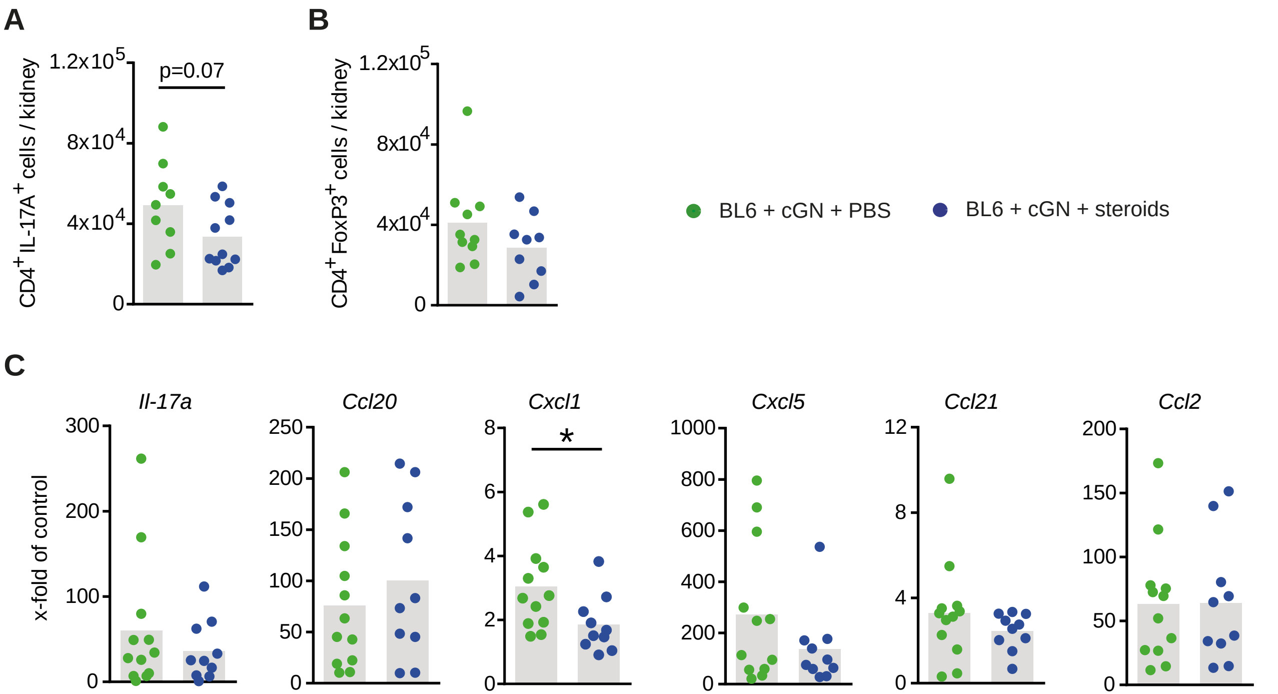

Supplemental Figure 9. Effects of glucocorticoid treatment on renal CD4$^+$ T-cell characteristics and renal cytokine mRNA and chemokine mRNA expression in murine cGN.

(**A**) Quantification of flow cytometric analyses of IL-17A production by CD3$^+$CD4$^+$ T cells isolated from kidneys of untreated and steroid-treated nephritic mice at day 10 of cGN. (**B**) Quantification of flow cytometric analyses of absolute numbers of CD3$^+$CD4$^+$FoxP3$^+$ Tregs performed for the previously mentioned groups. (**C**) RT-PCR analyses of indicated mRNA expression from whole renal cortices of kidneys from untreated and steroid-treated nephritic mice at day 10 of cGN. Data are representative of three independent experiments. Symbols represent individual data points, with the mean as a bar graph. Data were analyzed using a two-tailed t test. *p<0.05.

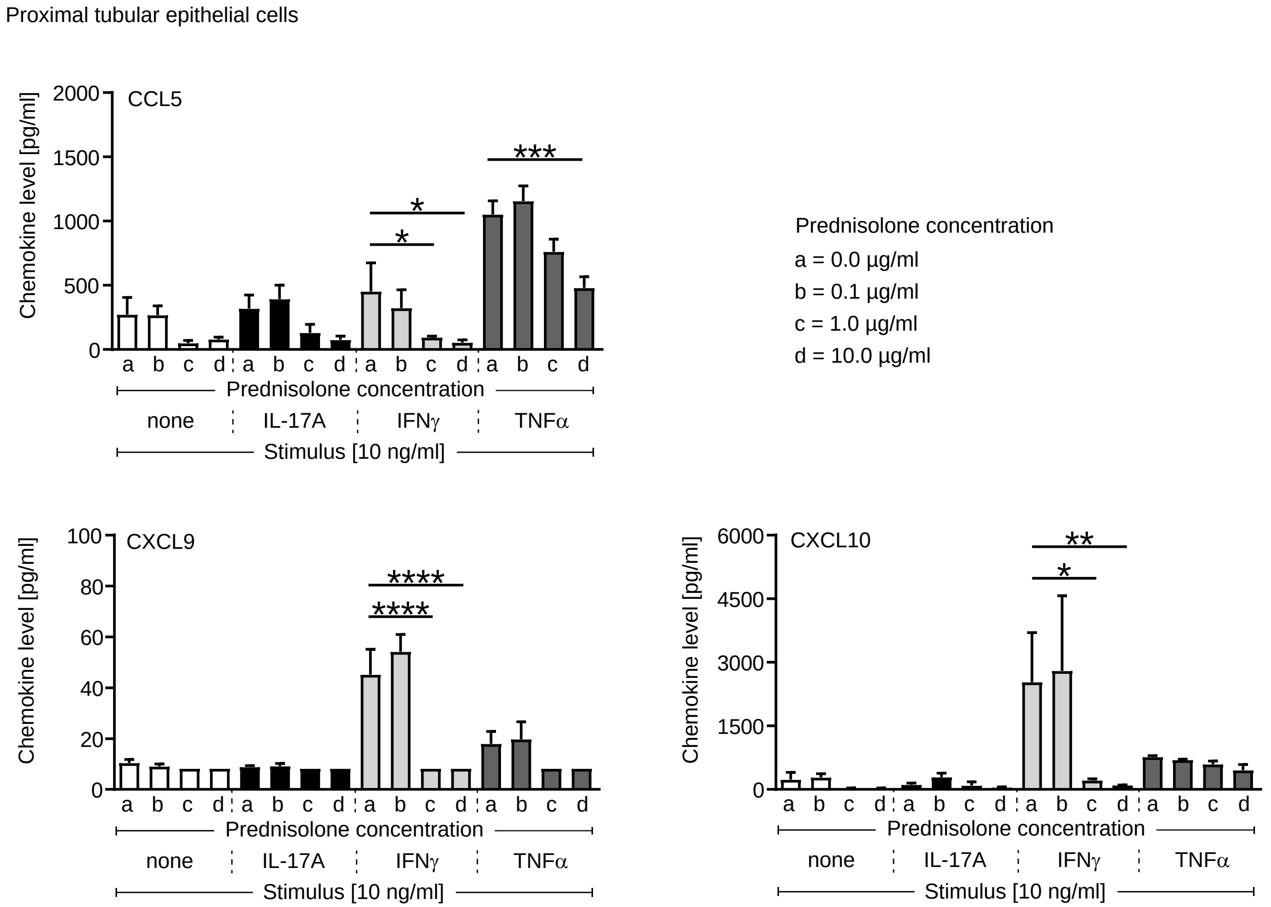

Supplemental Figure 10. Glucocorticoids reduce cytokine-induced production of T_H1-associated chemokines by proximal tubular epithelial cells in a dose-dependet manner.

Chemokine protein levels in supernatants of proximal tubular epithelial cells after stimulation with medium alone, IL-17A, IFNγ, or TNFα under increasing concentrations of prednisolone. Data are presented as bar graphs with the mean and standard deviation. Data were analyzed using a two-way ANOVA with Bonferroni's multiple comparisons test. *p<0.05, **p<0.01, ***p<0.001, ****p<0.0001.

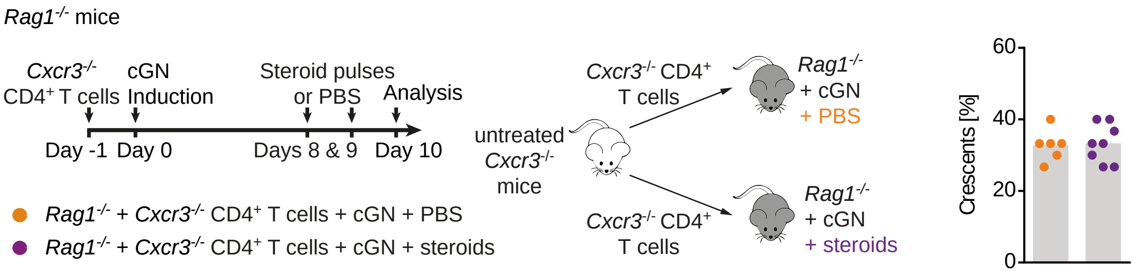

Supplemental Figure 11. Renal crescent formation in nephritic *RAG1*$^{-/-}$ mice with or without glucocorticoid treatment after transfer of CXCR3-deficient CD4^{+} T cells.

Schematic representation of the experimental setup and quantification of crescent formation of nephritic *Rag1*$^{-/-}$ mice treated with PBS or steroids two days before analysis and eleven days after intravenous transfer of CXCR3^{neg} CD4^{+} T cells into both groups. Symbols represent individual data points, with the mean as a bar graph. Data were analyzed using a two-tailed t test.

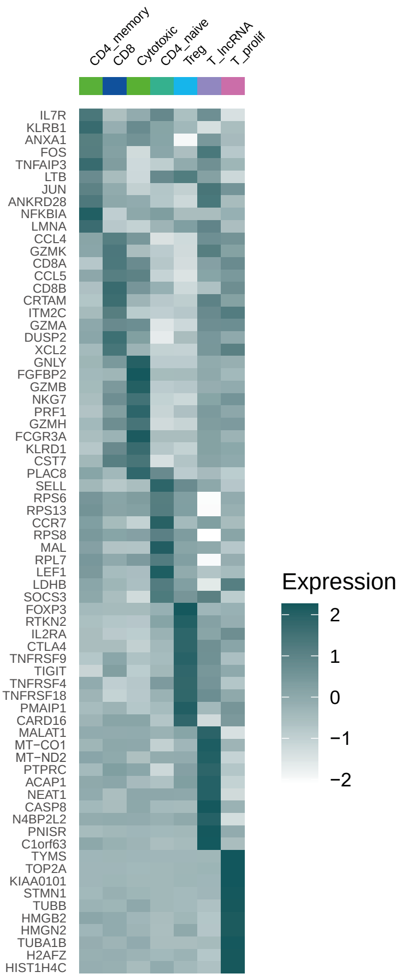

Supplemental Figure 12. Cluster-defining genes of human single-cell RNA sequencing.

Cluster-defining genes of seven clusters defined by unsupervised clustering of renal CD3^{+} T cells from kidney biopsies of ANCA-GN patients treated with glucocorticoids.
